# Healthcare use before paediatric multiple sclerosis onset differs by age and sex: a nationwide cohort study

**DOI:** 10.1136/bmjno-2025-001363

**Published:** 2025-12-23

**Authors:** Fredrik Sandesjö, Kyla McKay, Ali Manouchehrinia, Feng Zhu, Ruth Ann Marrie, Helen Tremlett

**Affiliations:** 1 Neuropaediatric Unit, Astrid Lindgren Children’s Hospital, Karolinska University Hospital, Stockholm, Sweden; 2 Department of Women's and Children’s Health, Karolinska Institutet, Stockholm, Sweden; 3 Department of Clinical Neuroscience, Karolinska Institutet, Stockholm, Sweden; 4 Center for Molecular Medicine, Karolinska University Hospital, Stockholm, Sweden; 5 Medicine (Neurology), University of British Columbia, Vancouver, British Columbia, Canada; 6 Department of Medicine, Dalhousie University Faculty of Medicine, Halifax, Nova Scotia, Canada

**Keywords:** MULTIPLE SCLEROSIS, EPIDEMIOLOGY, PAEDIATRIC

## Abstract

**Background:**

Increased use of healthcare before multiple sclerosis (MS) onset indicates a prodromal phase. Data on the prodromal phase of paediatric-onset MS (PoMS) are sparse, especially regarding age-specific and sex-specific characteristics. The objective of this study was to examine healthcare use by age and sex before PoMS clinical onset.

**Methods:**

Individuals with MS with symptom onset before age 18 were identified in the Swedish MS registry. Rates of hospitalisations, outpatient visits and prescriptions dispensed were compared with a matched non-MS cohort for up to 16 years before MS onset, categorised by age at onset (12–15 and 16–17 years) and sex.

**Results:**

We included 233 PoMS and 1151 matched individuals. Compared with the matched cohort, females with PoMS exhibited higher annual rates of outpatient visits in the years preceding MS onset for neoplasms (rate ratios (RRs) 3.44–5.73) while males had higher rates for skin-related visits (RRs 6.00–11.00) and prescription dispensations for corticosteroids for dermatological use (year −1, RR 3.12; 95% CI 1.01 to 9.68). Older teenagers with MS had higher visit rates for neoplasms (RRs 3.98–8.70), while younger teenagers had higher nervous system-related visit rates (RRs 4.51–5.97) before MS onset.

**Conclusions:**

Individuals with PoMS showed age-specific and sex-specific increases in healthcare use before symptom onset, including skin-related visits in males and neoplasm-related visits for females and 16–17 year-olds. These results can help guide our understanding of MS mechanisms and may aid in earlier detection of MS in the paediatric population.

**WHAT IS ALREADY KNOWN ON THIS TOPIC**

- A prodromal phase with increased healthcare use has been described in adult-onset multiple sclerosis (MS), but evidence in paediatric-onset MS (PoMS) is limited. Prior studies have not systematically explored how these patterns vary by age and sex.

**WHAT THIS STUDY ADDS**

- This study reveals distinct age-specific and sex-specific patterns of healthcare use prior to PoMS onset. Males showed increased dermatological visits and corticosteroid use, while females and older adolescents had more neoplasm-related visits. Younger adolescents had elevated nervous system-related visits.

**HOW THIS STUDY MIGHT AFFECT RESEARCH, PRACTICE OR POLICY**

- Recognising age-specific and sex-specific healthcare patterns may improve understanding of early disease manifestations in PoMS. This may inform future research into MS pathogenesis and contribute to earlier identification and intervention strategies.

## Introduction

 Multiple sclerosis (MS) is a chronic neurological condition^1^ that affects an estimated 2.8 million people worldwide.^2^ While MS is typically diagnosed in adulthood, about 5% of individuals with MS experience the onset of symptoms in childhood or adolescence.^3^ Persons with paediatric-onset MS (PoMS) experience higher relapse rates^3^ and a greater MRI-lesion burden^4^ compared with the adult-onset MS population. Early treatment following MS symptom onset has shown beneficial results in minimising longer-term disability in PoMS.^5^ Thus, early recognition to facilitate prompt effective treatment initiation is crucial.^6^

Growing evidence has indicated that there is a prodromal phase preceding the classic symptom onset in MS.^7^ In the overall MS population, this has been observed most consistently through a rise in health service utilisation in the years before the disease is clinically identified.^8^,^10^ Further, evidence of biological markers of axonal damage (increased serum neurofilament light levels) supports the notion that the biological processes of MS begin years before their recognition.^11^ Recent evidence supports a prodromal phase in children as well, measurable as higher healthcare visits prior to MS recognition.^12^,^14^

Certain characteristics of the MS prodrome have been identified as predominantly affecting certain age groups or sexes in the general MS population.^15 16^ For instance, before MS onset, pain is more common in older adults and anaemia more common in men.^15^ It is unknown whether age-specific and sex-specific characteristics of the MS prodrome exist in the paediatric population. To address this, we conducted a population-based study in Sweden, stratifying our analysis of healthcare contacts in the pre-disease period by age group and sex. Understanding potential differences in prodromal features is crucial for improving early recognition and diagnosis of PoMS, which may ultimately enable earlier intervention and better long-term outcomes for affected children.

## Methods

### Setting, data sources and study population

Sweden has a universal, publicly funded healthcare system.^17^ All residents of Sweden have a unique personal identification number, allowing for linkage across multiple data platforms. Nationwide, population-based health administrative data, including information on hospital and outpatient visits and prescriptions dispensed, were available from the National Patient Register^18^ and Prescribed Drug Registry.^19^ Demographic information, including sex, date of birth and death, immigration/emigration status and county of residence, was obtained from the Total Population Register.^20^ Annualised family income was obtained from the Longitudinal Integrated Database for Health Insurance and Labour Market Studies.^21^ The onset date of MS symptoms, as recorded by a neurologist, was obtained from the Swedish MS Registry, a national quality register that captures an estimated 85% of Sweden’s prevalent MS population.^22^ Data from all sources were available from 1 January 2001 to 31 December 2019, except for prescription drug data, which were available from 1 January 2005.

The cohort included persons with a definite diagnosis of MS registered in the Swedish MS registry. The index date was defined as either the date of the first MS symptom as transcribed by a neurologist or the date of the first recorded central nervous system (CNS)-related demyelinating International Classification of Diseases (ICD) code (ICD-9-SE: 340, 377D, 323X, 341A, 341W, 341X, or ICD-10-SE: G35, H46-H48, G37.3, G36) recorded in the National Patient Register, whichever occurred first. Only individuals with an index date occurring before age 18 years were included.

Each person with PoMS was matched with up to five individuals from the general population who had no CNS demyelinating ICD codes listed in the National Patient Register. The matching was based on sex, year of birth, county of residence at the index year and duration of residency in Sweden before the index date (all persons were required to have a minimum of 5 years residency pre-index date). The individuals in the matched cohort had to reside in Sweden until their corresponding person with MS was diagnosed with MS and registered in the MS registry. We selected up to five people without MS per PoMS participant, a priori, to strike a balance between enhancing statistical power and maintaining efficiency.

### Outcomes and statistical methods

Three outcomes were compared between the PoMS and matched cohorts: hospitalisations, outpatient visits and prescriptions dispensed. Analyses were stratified by sex and age group (12–15, and 16–17, representing early and late childhood/adolescence) at the index date and measured in the period before the index going back to birth, if possible. Due to small numbers, the <12 age group was not included in the age-stratified analyses.

The reasons for healthcare use were analysed as rates of outpatient visits and hospitalisations grouped by primary ICD-10 chapter (online supplemental table 1). Prescription dispensation rates were grouped according to WHO’s Anatomical Therapeutic Chemical classification system, second level (therapeutic drug class) (online supplemental table 2). Outpatient visits and prescriptions dispensed were measured annually prior to the index date. Due to the low number of hospitalisations, they were assessed over a combined 5-year period before the index date. Comparisons were performed using quasi-Poisson regression models. To account for variable residency time in each year, the natural log of residence time was included as a model offset. Results were presented as rate ratios and 95% CIs. We only analysed periods (1 or 5 years) with at least 10 events in total among the PoMS group and the matched cohort. Analyses were conducted using R (V.4.0.5, R Foundation for Statistical Computing, Vienna, Austria).

## Results

### Cohort characteristics

The cohort comprised 233 persons with PoMS and 1151 matched individuals. The MS registry contained information on the symptom onset date for 226 (97%) persons with PoMS. The first recorded CNS demyelinating event in the National Patient Register was used as the index date for the seven persons with PoMS (3%) with a missing onset date. Additionally, among 14 persons with PoMS (6%), their first CNS demyelinating event occurred before their recorded symptom onset and was used as the index. Females predominated, and the median age at the index was 16.5 years. Demographic characteristics are available in table 1 and online supplemental table 3.

**Table 1:** Characteristics of the cohort consisting of 233 individuals with paediatric-onset multiple sclerosis, with symptom onset between 2001 and 2019, and 1151 persons in the matched cohort

	Paediatric-onset multiple sclerosis	Matched cohort
N	233	1151
Sex, N (%)		
Female	163 (70.0)	801 (69.6)
Male	70 (30.0)	350 (30.4)
Age at index in years		
Mean (SD)	15.9 (1.9)	16.0 (1.8)
Median (Q1, Q3)	16.5 (15.2, 17.3)	16.6 (15.1, 17.3)
Age at index (categories) in years, N (%)		
<12	10 (4.3)	44 (3.8)
12–15	43 (18.5)	233 (20.2)
16–17	180 (77.3)	874 (75.9)
Socioeconomic status, N (%)*		
1 (lowest income quintile, least affluent)	23 (9.9)	66 (5.7)
2	28 (12.0)	95 (8.3)
3	38 (16.3)	169 (14.7)
4	52 (22.3)	310 (26.9)
5 (highest income quintile, most affluent)	92 (39.5)	511 (44.4)
Immigrant status, N (%)		
Immigrant	13 (5.6)	36 (3.1)
Non-immigrant	220 (94.4)	1115 (96.9)
Area of residence, N (%)†		
Rural	59 (25.3)	287 (24.9)
Urban	174 (74.7)	864 (75.1)

* Income of the highest-earning parent. 14 matched individuals with missing parental socioeconomic status were assigned to category 3.

† In seven individuals with multiple sclerosis, information on residential area was missing and the residential area of the mother was used.

### Rates of hospital and outpatient physician visits

#### Sex-stratified

In the combined 5 years before the index year, females with PoMS had a higher rate of hospitalisations for endocrine and metabolic disorders than females in the matched cohort (rate ratio 5.73; 95% CI 1.53 to 21.49); the number of events among males was <10. No other sex-specific differences were observed for hospitalisations (data not shown).

In yearly analyses, compared with females in the matched cohort, females with PoMS had higher outpatient visit rates related to neoplasms (significant in pre-index years −1 to –2, and −4), sense organs (eg, eyes, ears, nose, tongue, skin), and ‘ill-defined signs and symptoms’ (eg, malaise, fatigue, sleep disturbances otherwise unspecified) (significant in year −1), and ‘other health system contact’ (eg, routine examinations, investigations, immunisations or counselling) (significant in years −1 and −15). These patterns were not observed among males with PoMS.

Among males with PoMS, compared with males in the matched cohort, the yearly analyses showed elevated visit rates for skin-related issues prior to the index (significant in years −1 and −2), as well as for ‘other health system contact’ (significant in year −1), the respiratory system (significant in years −2 and −12), injuries (significant in years −6 and −8) and visit rates for which the physician could not (or did not) assign an ICD code (significant in years −8 to –9, −10 and −12). These patterns were observed only among males (table 2 and onlinesupplemental figures S1  S2).

**Table 2:** Annual number of outpatient visits, categorised by ICD chapters, for individuals with PoMS and matched individuals in Sweden from 2001 to 2019

Strata	Year*	ICD chapter		Rate ratios (95% CIs)	Outpatient visits among individuals with PoMS/matched individuals†
Female	–1	2	Neoplasms	3.44 (1.05 to 11.28)	7/10
Female	–1	7	Sense organs (eg, eyes, ears, nose, tongue, skin)	4.39 (1.86 to 10.37)	25/28
Female	–1	17	Ill-defined signs, symptoms	3.86 (2.34 to 6.38)	44/56
Female	–1	19	Other health system contact	3.06 (1.80 to 5.22)	48/77
Female	–2	2	Neoplasms	5.73 (1.09 to 30.02)	7/6
Female	–4	2	Neoplasms	5.41 (1.71 to 17.09)	11/10
Female	−15	19	Other health system contact	4.54 (1.26 to 16.37)	5/7
Male	–1	13	Skin-related	11.00 (2.74 to 44.14)	11/5
Male	–1	19	Other health system contact	2.62 (1.16 to 5.90)	11/21
Male	–2	9	Respiratory system	3.33 (1.05 to 10.54)	8/12
Male	–2	13	Skin-related	6.00 (1.68 to 21.38)	6/5
Male	–6	18	Injury-related	2.44 (1.16 to 5.10)	14/29
Male	–8	18	Injury-related	3.61 (1.60 to 8.17)	13/19
Male	–8		Unassigned ICD code	5.94 (2.04 to 17.30)	27/24
Male	–9		Unassigned ICD code	11.26 (2.97 to 42.66)	32/15
Male	−10		Unassigned ICD code	10.50 (3.20 to 34.39)	28/14
Male	−12	9	Respiratory system	3.72 (1.33 to 10.44)	10/14
Male	−12	20	Unassigned ICD code	3.91 (1.17 to 13.08)	6/8
12–15 years	–1	6	Nervous system	4.51 (1.51 to 13.47)	11/12
12–15 years	–1	17	Ill-defined signs, symptoms	3.07 (1.41 to 6.68)	15/24
12–15 years	–7	6	Nervous system	5.97 (1.53 to 23.24)	6/5
12–15 years	−12	18	Injury-related	4.09 (1.16 to 14.41)	5/6
16–17 years	–1	2	Neoplasms	3.98 (1.26 to 12.53)	8/10
16–17 years	–1	7	Sense organs (eg, eyes, ears, nose, tongue, skin)	7.15 (2.75 to 18.54)	23/16
16–17 years	–1	17	Ill-defined signs, symptoms	3.42 (1.91 to 6.11)	33/48
16–17 years	–1	19	Other health system contact	3.69 (2.05 to 6.62)	43/58
16–17 years	–3	2	Neoplasms	8.70 (1.92 to 39.50)	7/4
16–17 years	–4	2	Neoplasms	5.47 (1.73 to 17.25)	11/10
16–17 years	–7	11	Genitourinary system	7.59 (1.17 to 49.01)	6/4
16–17 years	–8	13	Skin-related	6.08 (1.17 to 31.63)	6/5
16–17 years	–8	18	Injury-related	2.54 (1.21 to 5.31)	16/32
16–17 years	–8		Unassigned ICD code	4.25 (1.59 to 11.38)	26/31
16–17 years	–9		Unassigned ICD code	7.86 (2.68 to 23.09)	38/25
16–17 years	−10		Unassigned ICD code	6.52 (2.18 to 19.46)	27/22
16–17 years	−12	9	Respiratory system	2.55 (1.06 to 6.12)	9/19
16–17 years	−13	19	Other health system contact	2.94 (1.15 to 7.51)	8/15
16–17 years	−16	1	Infection-related	5.34 (1.56 to 18.20)	5/5

The data are stratified by sex and age at the index date, highlighting only the results with significant differences between the strata. Included are rate ratios, 95% CIs and p values.

* Preceding the index date.

† Matched up to 5:1. Ill-defined signs and symptoms refer to symptoms, signs (eg, malaise, fatigue, sleep disturbances otherwise unspecified), abnormal results of clinical or other investigative procedures and ill-defined conditions for which no diagnosis classifiable elsewhere is recorded. Unassigned ICD codes refer to outpatient visits for which a physician either could not or did not assign an ICD code. Other health system contact refers to occasions when circumstances other than a disease, injury or external cause classifiable as other ICD chapters were recorded, for instance, routine examinations, investigations, immunisations or counselling.

ICD, International Classification of Diseases; PoMS, paediatric-onset multiple sclerosis.

#### Age-stratified

There were no significant differences in ICD-chapter-specific hospitalisations in the 5-year pre-index period between cohorts in either age category.

Among individuals with PoMS with an MS onset between the ages of 12–15, compared with the same age group in the matched cohort, the yearly analyses showed higher outpatient visit rates related to the nervous system pre-index (significant in years −1 and −7, a pattern not seen among patients with onset between 16–17), ‘ill-defined signs and symptoms’ (significant in year −1) and injuries (significant in year −12).

Among individuals with PoMS with an index date between the ages of 16–17, the yearly analyses showed higher visit rates related to neoplasms pre-index (significant in years −1 to –3 and −4) compared with the same age group in the matched cohort. Further, visit rates related to sense organs, ‘ill-defined symptoms and signs’, ‘other health system contact’, the genitourinary system, injuries, the respiratory system, infections and visits with unassigned ICD codes were intermittently higher in the 1 to 16 years before the index date. Most of these patterns were not observed among persons aged 12–15 years at the index date (table 2 and onlinesupplemental figures S3  S4).

### Prescription drug classes dispensed

The age and sex-stratified analyses showed generally higher rates of specific prescriptions dispensed among persons with PoMS compared with the non-MS group during seven of the ten years leading up to the index date. There was no evidence of a statistically lower rate of prescriptions dispensed to persons with PoMS in any year.

#### Sex-stratified

Among females with PoMS, compared with females in the matched cohort, the prescription rates for gynaecological medications were higher in the year before the index date, as were rates for ophthalmological and otological preparations (significant in year −2), and sex hormones and genital system modulators (significant in year −4). These elevations were not observed among males.

In males with PoMS, compared with males in the matched cohort, the rates of corticosteroid prescriptions for dermatological use were higher in the year pre-index, with nasal preparations and medications for obstructive airway diseases being elevated in years five and six pre-index, respectively. These elevations were not observed among females.

#### Age-stratified

Among persons with PoMS aged 16–17 at the index date, prescription rates for drugs related to obstructive airway diseases were higher (significant in year −5), cough and cold medications and systemic antibiotics (significant in year −7) and corticosteroids for dermatological use (significant in year −10) before the index date. No differences in prescription rates were observed among persons aged 12–15 at the index (table 3).

**Table 3:** Annual number of prescriptions dispensed, categorised by therapeutic drug class (Anatomical Therapeutic Chemical Classification, second level), for individuals with PoMS and matched individuals in Sweden from 2005 to 2019

Strata	Year*	Anatomical Therapeutic Chemical Classification, second level		Rate ratios (95% CIs)	Outpatient visits among individuals with PoMS/matched individuals†
Female	–1	G02	Other gynaecologicals	4.05 (1.03 to 15.90)	5/5
Female	–2	S03	Ophthalmological and otological preparations	3.47 (1.07 to 11.25)	5/7
Female	–4	G03	Sex hormones and modulators of the genital system	3.24 (1.15 to 9.09)	8/12
Male	–1	D07	Corticosteroids for dermatological use	3.12 (1.01 to 9.68)	5/8
Male	–5	R01	Nasal preparations	3.75 (1.13 to 12.39)	6/8
Male	–6	R03	Drugs for obstructive airway diseases	3.38 (1.25 to 9.13)	8/12
16–17 years	–5	R03	Drugs for obstructive airway diseases	2.67 (1.25 to 5.71)	14/26
16–17 years	–7	R05	Cough and cold preparations	2.34 (1.12 to 4.87)	12/26
16–17 years	–7	J01	Systemic antibiotics	1.88 (1.07 to 3.31)	26/70
16–17 years	−10	D07	Corticosteroids for dermatological use	5.31 (1.51 to 18.73)	5/5

The data are stratified by sex and age at the index date, highlighting only the results with significant differences (p<0.05) between the strata.

* Preceding the index date.

† Matched up to 5:1.

PoMS, paediatric-onset multiple sclerosis.

## Discussion

In this population-based cohort study, comprising 233 persons with PoMS and 1151 without, we identified sex and age-related differences in healthcare use in the years before the index date (MS symptom onset). Reasons for healthcare use varied, but there were measurable differences between the sex and age-stratified PoMS groups and the matched cohorts up to 16 years prior to the index. Most consistently, outpatient visits for neoplasms were elevated in females and older adolescents, while skin-related visits and dermatological prescriptions, and injury-related visits were elevated in males with PoMS before MS onset. Only among persons with an MS onset between ages 12–15 were visits for the nervous system elevated before MS onset. Females with PoMS had higher rates of gynaecological medications, sex hormones and genital system modulators compared with females without MS, in the year before the index.

Few studies have explored sex-related or age-related differences in the PoMS prodrome (before MS symptom onset).^14^ A population-based study from Germany accessed primary care data and identified increased visits for metabolic disorders, including obesity, prior to medical recognition of MS, measured using ICD codes in administrative data.^12^ However, authors were unable to access the (clinical) date of MS symptom onset or data beyond that captured in primary care. We found increased hospitalisations for endocrine and metabolic disorders in females only during the 5-year period preceding the index date (MS symptom onset). Thyroid disorders, obesity and type I diabetes are contained in this chapter. Thyroid disorders are more commonly comorbid among people with MS compared with people without.^23^ Obesity has been identified as a risk factor for MS in adolescent females, aligning with our results.^24^ Type 1 diabetes, particularly in children, often begins with hospitalisation, which could also have contributed to this finding. Although human leucocyte antigen patterns of diabetes and MS are generally considered mutually exclusive, there is evidence of an increased risk of MS among persons with type 1 diabetes.^25^ In addition to the observed endocrine associations, previous studies have also reported links between MS and other autoimmune conditions, including inflammatory bowel disease^26^ and rheumatoid arthritis^27^ after MS onset. We did not observe such associations in the pre-disease period, which may be in part because we focused our analyses to the ICD chapter level.

A Canadian study reported increased visits to the dermatologist in adults with relapsing MS in the 5 years before MS onset compared with the matched cohort.^28^ As there is evidence of a higher incidence of immune-mediated skin diseases in patients with MS,^29 30^ the authors suggest that skin diseases may be an early sign of immune dysregulation.^28^ We found males to have an 11-fold increase in outpatient visits for skin-related disorders in the year preceding the index, and a 6-fold increase 2 years before. Moreover, males had a higher number of prescriptions dispensed for corticosteroids for dermatological use in the year prior to the index. While event numbers were small, findings raise the intriguing possibility that there may be alterations in the skin microbiome or skin-related immune dysregulation during the MS prodrome that disproportionately affects males. The findings could also relate to differences in help-seeking behaviours, as adolescent males tend to delay seeking healthcare, which could lead to greater severity in disease^31^ and could have contributed to their use of specialised outpatient services in greater numbers than females.

During the years preceding the index date, females and older teens had increased specialist visits related to neoplasms. Increased visit rates due to neoplasms have been previously documented in administrative data for adults,^8^ and recently in PoMS as well. In fact, in Ontario, Canada, hospitalisations for neoplasms were over four times higher over the full pre-index period in MS cases relative to the matched non-MS cohort.^14^ Chronic inflammation is an established risk factor for cancer,^32^ and there is a possibility that the inflammation occurring before the clinical onset of MS may also contribute to cancer development. Alternatively, cancer therapies can induce autoimmunity^33^ and may have contributed to the triggering of MS onset. Last, it is also possible that MS was incidentally diagnosed during the diagnostic work-up or monitoring of CNS tumours.

Further, we observed elevated rates of respiratory system-related visits and prescriptions for drugs targeting obstructive airway diseases before MS onset in males and individuals aged 16–17 years at the index. Specific reasons for these visits and prescriptions could include chronic or acute respiratory illness, such as asthma or pneumonia, each of which has been shown to be associated with MS before onset or diagnosis in prior studies.^1234^,^36^ We observed increased injury-related visits among males 6–8 years before MS onset. A Swedish study examined the incidence of concussions and leg and arm fractures before MS diagnosis. The findings indicated that while concussion was associated with an increased risk of MS, fractures were not, suggesting that the risk is specifically related to head injuries rather than general trauma.^37^ There are studies both supporting^37 38^ and dismissing^39^ an association between adolescent concussions and subsequent MS development. In a Canadian study, an increased risk of MS was observed after concussions during adolescence among males.^38^ Although we lack data on specific ICD codes, beyond chapter level, and cannot determine if the males in our study experienced head trauma or other kinds of trauma, our findings give some support to the theory that trauma may be a risk factor for MS.

We noted elevated visit rates associated with the nervous system in persons with PoMS aged 12–15 at onset compared with the same age group in the matched cohort. This pattern, exclusively seen in this stratum, was noted during the 7 years prior to the index, reaching significance in years −1 and −7. It seems reasonable that the symptoms presented during these visits may have been early signs of MS, which could have potentially led to an earlier diagnosis. The reason this phenomenon would be observed in the younger group may result from the fact that MS is less prevalent^40^ and therefore less well-recognised in this age group.

Finally, males with PoMS and persons with PoMS in the older age stratum had more outpatient visits with unassigned ICD codes in the 8–12 years before the index date. While it is unclear why codes are missing, it may indicate an unspecific clinical picture, which is hard to characterise with a specific ICD code. If so, these visits could, hypothetically, be due to symptoms of the ‘paediatric prodrome’, in line with findings of symptoms of an unspecific nature.^12^

### Strengths and limitations

This was a nationwide registry-based cohort study that, unlike most previous studies on the topic, accessed data on the clinical onset of MS as recorded by a neurologist. The clinical onset is closer to the true onset of MS than administrative definitions, which can only capture medical recognition of the disease. Therefore, using data on the clinical onset allows for a more precise evaluation of the ‘true’ prodrome. Another strength of this study is the paediatric study population. As these individuals are younger, they have experienced fewer negative health behaviours and developed fewer comorbidities than adults. This provides a greater chance to detect early indicators of disease.

This study carries the risk of both type I and II errors. The limited number of events in certain analyses meant that we could not run models for each ICD chapter or prescription class in every pre-index year. Similarly, we were unable to explore more granular reasons for healthcare encounters below the ICD chapter/ATC second level. Conversely, because we conducted multiple analyses, there is an increased risk of incorrectly identifying an association. We lacked information on symptoms that did not lead individuals to pursue healthcare, such as mild, self-managed or spontaneously resolved symptoms. Finally, we lacked data from primary care providers. As general practitioners act as the most common initial point of entry to healthcare, some early symptoms of the PoMS prodrome may have been missed. This also had the effect of reducing event rates and thereby reducing statistical power.

## Conclusions

Our findings show that individuals with PoMS exhibited age-specific and sex-specific increases in healthcare utilisation before the onset of MS than those without MS. These results deepen our understanding of the underlying mechanisms of MS, facilitating earlier detection, improved management and, ultimately, the prevention of PoMS.

## Supplementary material

10.1136/bmjno-2025-001363 online supplemental figure 1

10.1136/bmjno-2025-001363 online supplemental figure 2

10.1136/bmjno-2025-001363 online supplemental figure 3

10.1136/bmjno-2025-001363 online supplemental figure 4

10.1136/bmjno-2025-001363 online supplemental table 1

10.1136/bmjno-2025-001363 online supplemental file 1

## Data Availability

Data are available upon reasonable request.
