# Supplementary figures and images for: Healthcare use before paediatric multiple sclerosis onset differs by age and sex: a nationwide cohort study
Source: BMJ Neurol Open. 2025 Dec 23;7(2):e001363. doi: 10.1136/bmjno-2025-001363 (PMC12750784; doi:10.1136/bmjno-2025-001363)

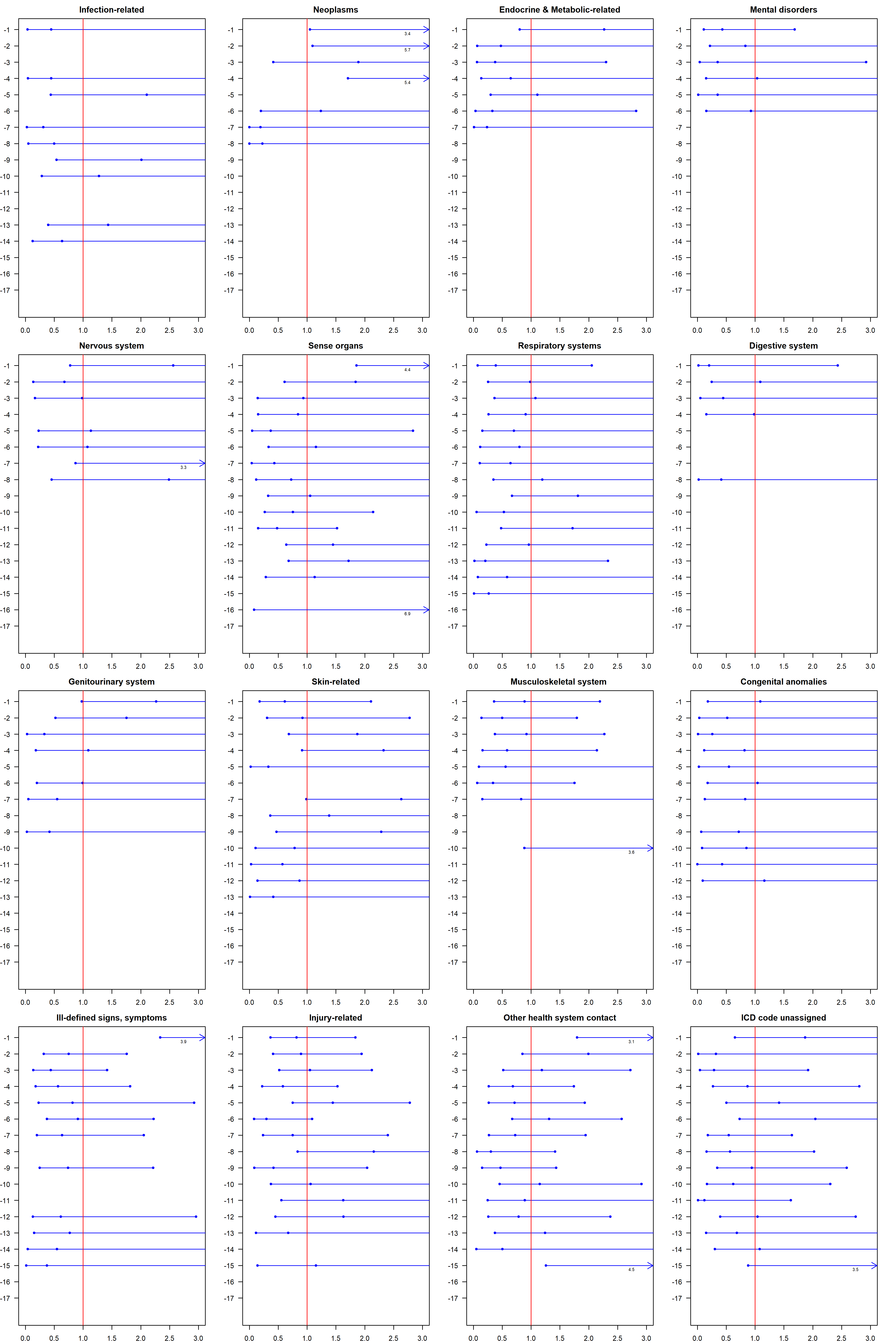

Supplement: online supplemental figure 1 [file bmjno-7-2-s001.tiff]

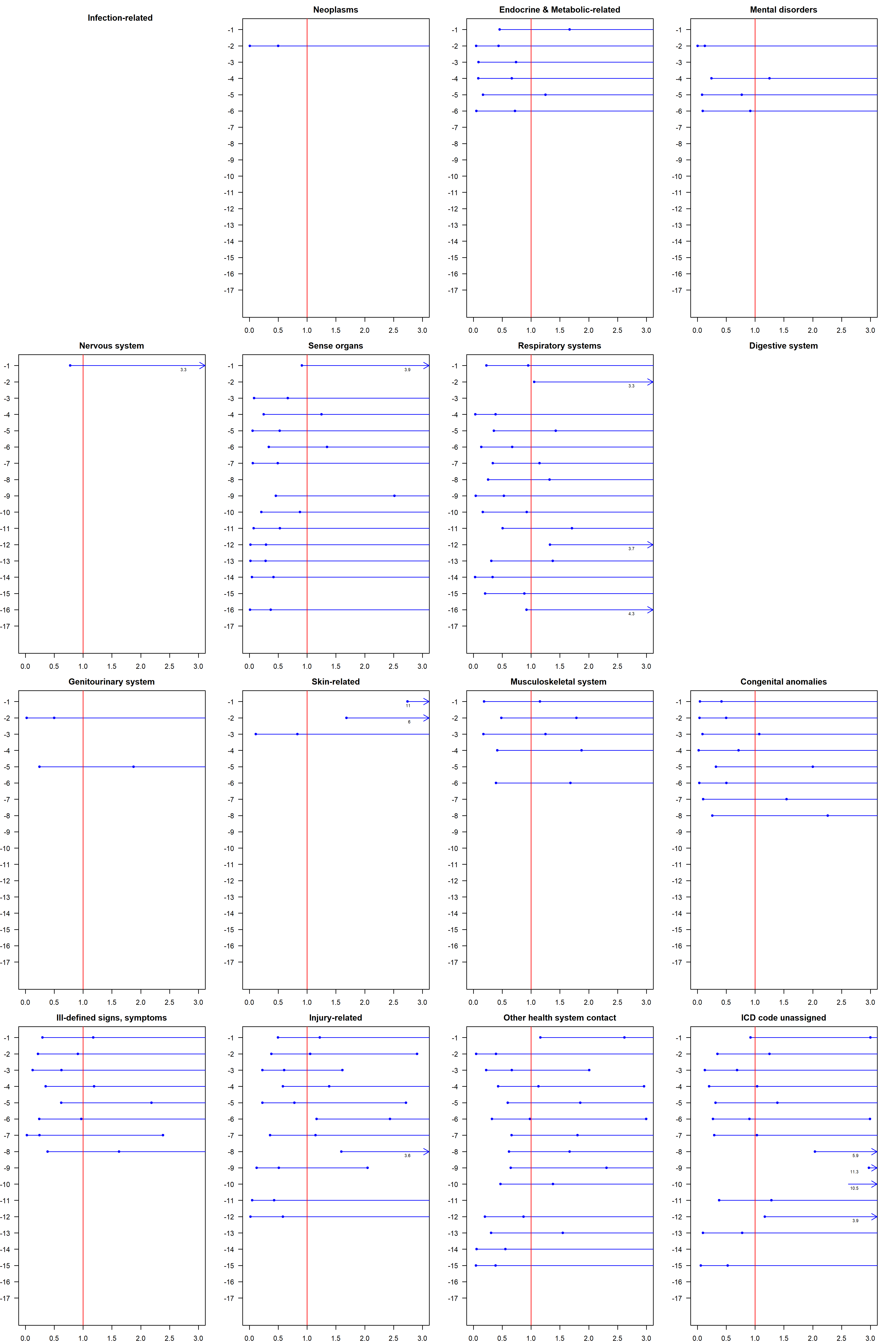

Supplement: online supplemental figure 2 [file bmjno-7-2-s002.tiff]

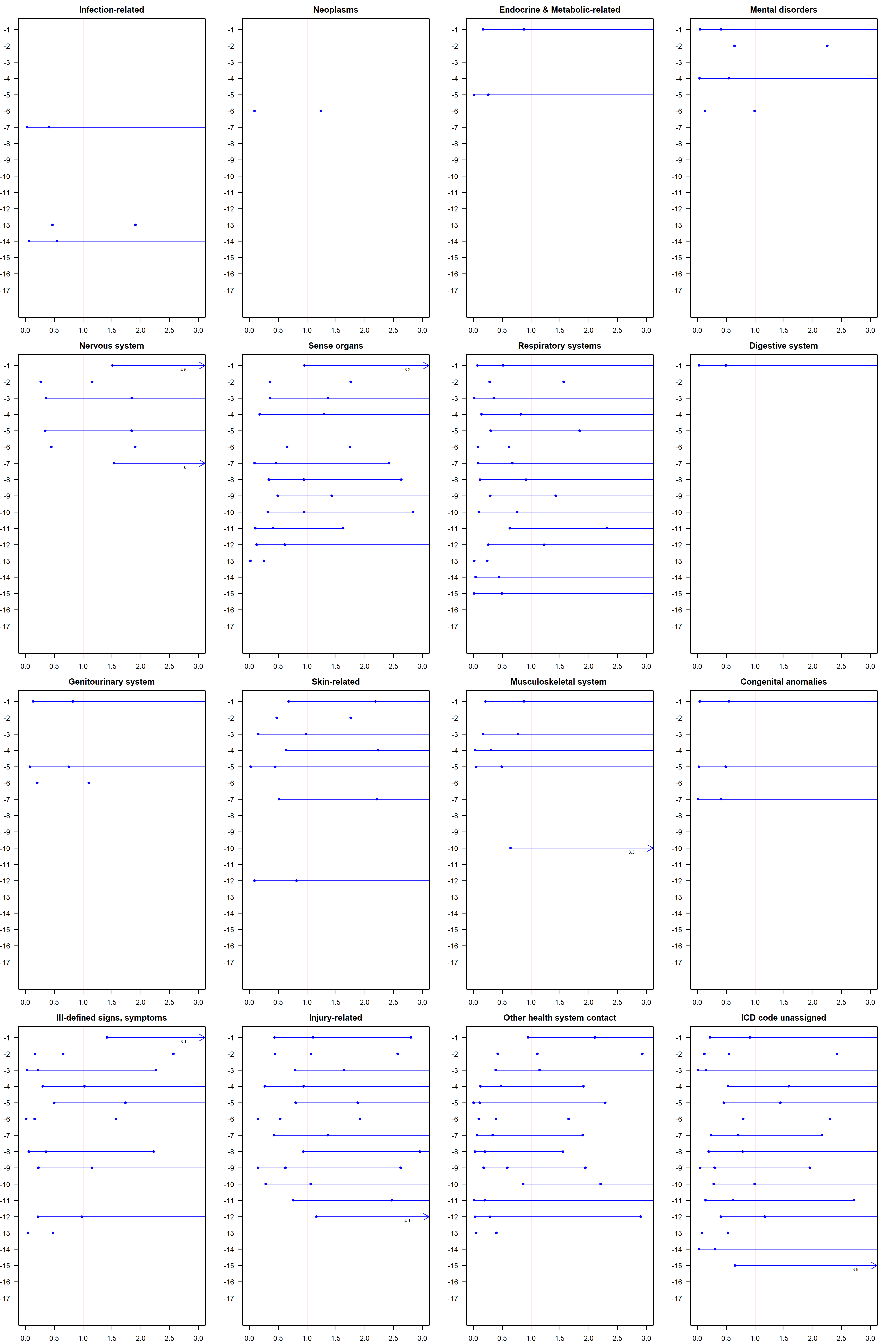

Supplement: online supplemental figure 3 [file bmjno-7-2-s003.tiff]

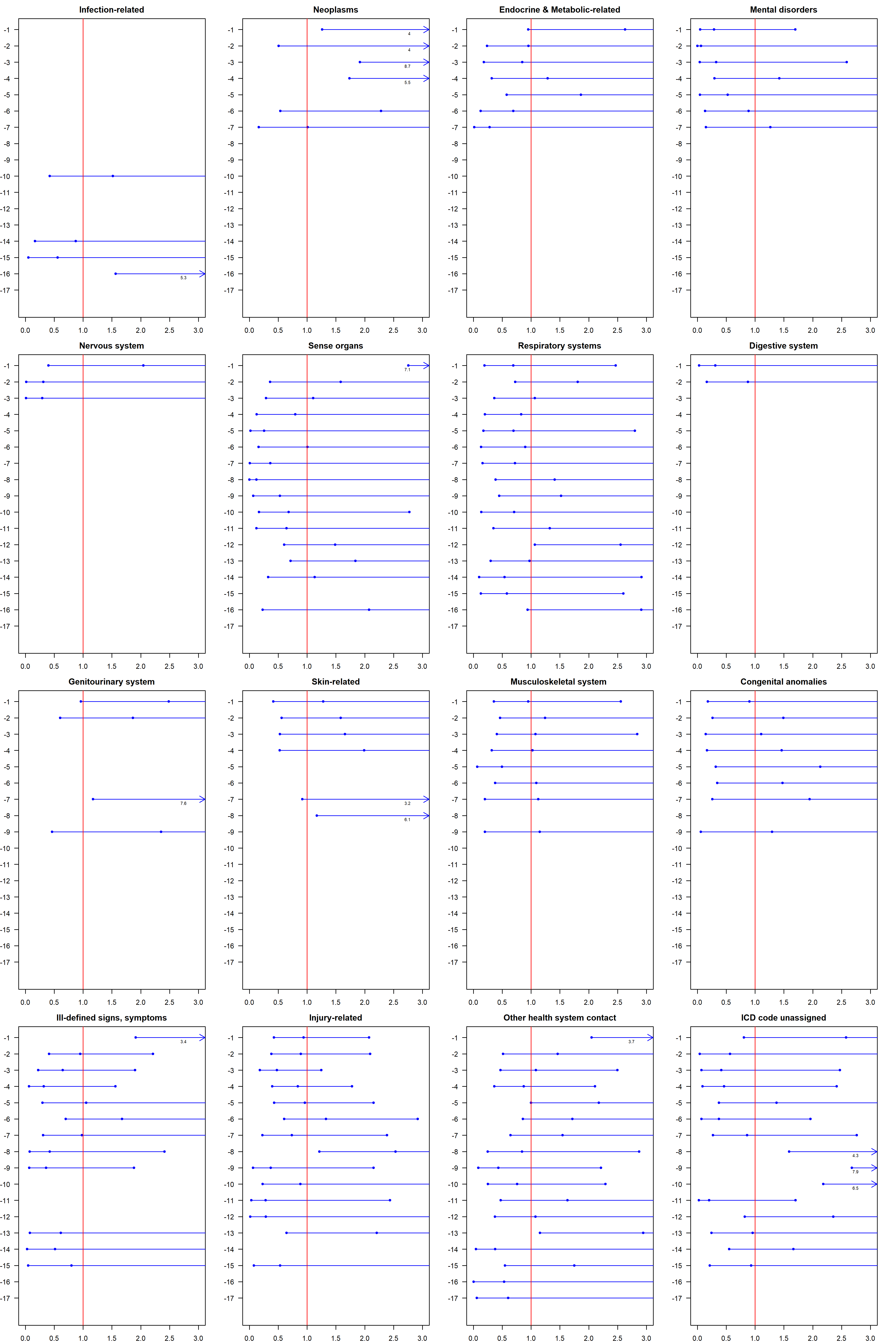

Supplement: online supplemental figure 4 [file bmjno-7-2-s004.tiff]
