# Supplementary material for: Healthcare use before paediatric multiple sclerosis onset differs by age and sex: a nationwide cohort study
Source: BMJ Neurol Open. 2025 Dec 23;7(2):e001363. doi: 10.1136/bmjno-2025-001363 (PMC12750784; doi:10.1136/bmjno-2025-001363)
Supplement: online supplemental table 1 [file bmjno-7-2-s005.docx]

|  | Pediatric-onset Multiple Sclerosis | Matched cohort |
| --- | --- | --- |
| N | 140 | 686 |
| **Sex N (%)** |  |  |
| Female | 97 (69.3) | 471 (68.7) |
| Male | 43 (30.7) | 215 (31.3) |
| **Age at Index. years** |  |  |
| Mean (SD) | 16.0 (1.8) | 16.0 (1.8) |
| Median (Q1. Q3) | 16.5 (15.1. 17.3) | 16.5 (15.0. 17.3) |
| **Age at Index (categories). years. N (%)** |  |  |
| <12 | 6 (4.3) | 27 (3.9) |
| 12-15 | 51 (36.4) | 248 (36.2) |
| 16-17 | 83 (59.3) | 411 (59.9) |
| **Socioeconomic status, N (%) *** |  |  |
| 1 (lowest income quintile, least affluent) | 17 (12.1) | 36 (5.2) |
| 2 | 20 (14.3) | 64 (9.3) |
| 3 | 23 (16.4) | 99 (14.4) |
| 4 | 31 (22.1) | 165 (24.1) |
| 5 (highest income quintile, most affluent) | 49 (35.0) | 322 (46.9) |
| **Immigrant status, N (%)** |  |  |
| Immigrant | 8 (5.7) | 14 (2.0) |
| Non-immigrant | 132 (94.3) | 672 (98.0) |
| **Area of residence, N (%) **** |  |  |
| Rural | 32 (22.9) | 152 (22.2) |
| Urban | 108 (77.1) | 534 (77.8) |

**Supplementary Table 1.** Characteristics of the cohort for the analysis of prescriptions dispensed. The cohort consists of 140 individuals with pediatric-onset multiple sclerosis with symptom onset between 2005 and 2019 and 686 persons in the matched cohort. SD: standard deviation.

| **No** | **ICD Chapters** | **Abbreviation** | **Diagnostic Codes** |
| --- | --- | --- | --- |
| 1 | Certain infectious and parasitic diseases | Infection-related | ICD-10: A00 – B99 |
| 2 | Neoplasms | Neoplasms | ICD-10: C00 – D49 |
| 3 | Endocrine. Nutritional. and Metabolic Diseases and Immunity Disorders | Endocrine & Metabolic-related | ICD-10: E00 – E89 |
| 4 | Diseases Of the Blood and Blood-Forming Organs | Blood-related | ICD-10: D50 – D89 |
| 5 | Mental. Behavioral and Neurodevelopmental disorders | Mental disorders | ICD-10: F01 – F99 |
| 6 | Diseases of the nervous system | Nervous system | ICD-10: G00 – G99 |
| 7 | Diseases of the sense organs | Sense organs | ICD-10: H00-H95 |
| 8 | Diseases of the circulatory system | Circulatory system | ICD-10: I00 – I99 |
| 9 | Diseases of the respiratory system | Respiratory systems | ICD-10: J00 – J99 |
| 10 | Diseases of the digestive system | Digestive system | ICD-10: K00 – K95 |
| 11 | Diseases of the genitourinary system | Genitourinary system | ICD-10:N00 – N99 |
| 12 | Pregnancy. childbirth and the puerperium | Pregnancy. childbirth | ICD-10: O00–O99 |
| 13 | Diseases of the skin and subcutaneous tissues | Skin-related | ICD-10: L00 – L99 |
| 14 | Diseases of the musculoskeletal system and connective tissues | Musculoskeletal system | ICD-10: M00 – M99 |
| 15 | Congenital anomalies | Congenital anomalies | ICD-10: Q00–Q99 |
| 16 | Certain conditions originating in the perinatal period | Perinatal-related | ICD-10: P00–P96 |
| 17 | Symptoms. Signs. and Ill-Defined Conditions | Ill-defined signs. symptoms | ICD-10: R00 – R99 |
| 18 | Injury and poisoning  Complication of surgical and medical care | Injury-related | ICD-10: S00 – T98  ICD-10: V00-Y99 |
| 19 | Factors Influencing Health Status and Contact with Health Services | Other health system contact | ICD-10: Z00 – Z99 |
| None | Missing or unassigned ICD Code | ICD code unassigned | None listed |

**Supplementary Table 2.** International Classification of Disease (ICD)-10 Chapters.

| **ATC 1^st^ Level** | **ATC 2^nd^ Level** | **ATC 2^nd^ Level Description** |
| --- | --- | --- |
| **A**: Alimentary Tract and Metabolism | A02 | Drugs for acid-related disorders |
|  | A06 | Drugs for constipation |
|  | A10 | Drugs used in diabetes |
| **B**: Blood and Blood Forming Organs | B03 | Anti-anemic agents |
| **D**: Dermatologicals | D01 | Antifungals for dermatological use |
|  | D02 | Emollients and protectives |
|  | D06 | Antibiotics and chemotherapeutics for dermatological use |
|  | D07 | Corticosteroids. dermatological preparations |
|  | D10 | Anti-acne preparations |
| **G**: Genito-Urinary System and Sex Hormones | G02 | Other gynecologicals |
|  | G03 | Sex hormones and modulators of the genital system |
| **H**: Systemic Hormonal Preparations. Excluding Sex Hormones and Insulins | H02 | Corticosteroids for systemic use |
| **J**: Antiinfectives for Systemic Use | J01 | Antibacterials for systemic use |
|  | J07 | Vaccines |
| **M**: Musculo-Skeletal System | M01 | Antiinflammatory and antirheumatic products |
| **N**: Nervous System | N02 | Analgesics |
|  | N03 | Antiepileptics |
|  | N05 | Psycholeptics |
|  | N06 | Psychoanaleptics |
| **P**: Antiparasitic Products | P02 | Anthelmintics |
| **R**: Respiratory System | R01 | Nasal preparations |
|  | R03 | Drugs for obstructive airway diseases |
|  | R05 | Cough and cold preparations |
|  | R06 | Antihistamines for systemic use |
| **S**: Sensory Organs | S01 | Ophthalmologicals |
|  | S03 | Ophthalmological and ontological preparations |

**Supplementary Table 3.** Anatomical Therapeutic Chemical (ATC) classification system 1^st^ and 2^nd^ levels. Only ATC 2^nd^ level therapeutic classes analyzed in at least one year are listed. Those with fewer than ten events among the pediatric-onset multiple sclerosis and matched cohort combined, where the statistical model did not run, or where the upper 95% confidence interval extended to infinity due to small number of events, were not analyzed or reported.
