# Supplementary material for: Healthcare use before paediatric multiple sclerosis onset differs by age and sex: a nationwide cohort study
Source: BMJ Neurol Open. 2025 Dec 23;7(2):e001363. doi: 10.1136/bmjno-2025-001363 (PMC12750784; doi:10.1136/bmjno-2025-001363)
Supplement: online supplemental file 1 [file bmjno-7-2-s006.docx]

**Supplementary Figure S1.** Outpatient visit rates among females with pediatric-onset multiple sclerosis (PoMS) compared to matched individuals, grouped by ICD-10 chapter. Rate ratios (RRs) and 95% confidence intervals are shown for each chapter, from the year prior to MS onset and annually back to birth, where data are available. An RR > 1 indicates a higher visit rate among individuals with PoMS compared to matched individuals.

**Supplementary Figure S2.** Outpatient visit rates among males with pediatric-onset multiple sclerosis (PoMS) compared to matched individuals, grouped by ICD-10 chapter. Rate ratios (RRs) and 95% confidence intervals are shown for each chapter, from the year prior to MS onset and annually back to birth, where data are available. An RR > 1 indicates a higher visit rate among individuals with PoMS compared to matched individuals.

**Supplementary Figure S3. Outpatient visit rates among individuals with** pediatric-onset multiple sclerosis (PoMS) **onset between** the ages of 12 and 15 **compared to matched individuals, grouped by ICD-10 chapter. Rate ratios (RRs) and 95% confidence intervals are shown for each chapter, from the year prior to MS onset and annually back to birth, where data are available. An RR > 1 indicates a higher visit rate among individuals with PoMS compared to matched individuals.**

**Supplementary Figure S4. Outpatient visit rates among individuals with** pediatric-onset multiple sclerosis (PoMS) **onset between** the ages of 16 and 17 **compared to matched individuals, grouped by ICD-10 chapter. Rate ratios (RRs) and 95% confidence intervals are shown for each chapter, from the year prior to MS onset and annually back to birth, where data are available. An RR > 1 indicates a higher visit rate among individuals with PoMS compared to matched individuals.**
